# Single-Step Synthesis of Highly Sensitive ^19^F MRI Tracers by Gradient Copolymerization-Induced Self-Assembly

**DOI:** 10.1021/acs.biomac.4c00915

**Published:** 2024-11-19

**Authors:** Vyshakh M. Panakkal, Dominik Havlicek, Ewa Pavlova, Klara Jirakova, Daniel Jirak, Ondrej Sedlacek

**Affiliations:** † Department of Physical and Macromolecular Chemistry, Faculty of Science, Charles University, 128 40 Prague 2, Czech Republic; ‡ Department of Diagnostic and Interventional Radiology, Institute for Clinical and Experimental Medicine, Videnska 1958/9, 140 21 Prague, Czech Republic; § Institute of Macromolecular Chemistry, v.v.i., Academy of Sciences of the Czech Republic, Heyrovsky Sq. 2, 162 06 Prague 6, Czech Republic; ∥ Institute of Biophysics and Informatics, First Faculty of Medicine, Charles University, Kateřinská 1660/32, 121 08 Prague, Czech Republic; ⊥ Third Faculty of Medicine, Charles University, Ruská 87, 100 00 Prague, Czech Republic; # Faculty of Health Studies, Technical University of Liberec, Studentská 1402/2, 46117 Liberec, Czech Republic

## Abstract

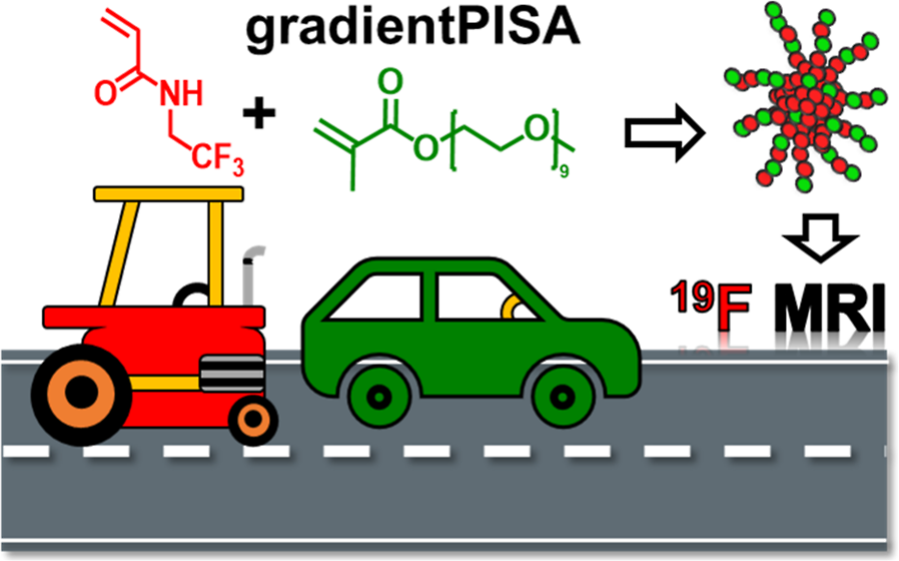

Amphiphilic gradient copolymers are promising alternatives to block copolymers for self-assembled nanomaterials due to their straightforward synthesis via statistical copolymerization of monomers with different reactivities and hydrophilicity. By carefully selecting monomers, nanoparticles can be synthesized in a single step through gradient copolymerization-induced self-assembly (gPISA). We synthesized highly sensitive ^19^F MRI nanotracers via aqueous dispersion gPISA of hydrophilic poly(ethylene glycol) methyl ether methacrylate (PEGMA) with core-forming *N*,*N*-(2,2,2-trifluoroethyl)acrylamide (TFEAM). The PPEGMA-grad-PTFEAM nanoparticles were optimized to achieve spherical morphology and exceptional ^19^F MRI performance. Noncytotoxicity was confirmed in Panc-1 cells. In vitro ^19^F MR relaxometry and imaging demonstrated their diagnostic imaging potential. Notably, these gradient copolymer nanotracers outperformed block copolymer analogs in ^19^F MRI performance due to their gradient architecture, enhancing ^19^F relaxivity. The synthetic versatility and superior ^19^F MRI performance of gradient copolymers highlight their potential in advanced diagnostic imaging applications.

## 1 Introduction

Polymerization-induced self-assembly (PISA) is a straightforward method to synthesize block copolymer nanoparticles.^1,2^ The discovery of PISA revealed new pathways to synthesize complex nanosystems, including access to various morphologies (e.g., spheres, worms, and vesicles), high nanoparticle concentrations, fast polymerization rates, and potentially easy industrial scale-up.^3^ PISA has been reported in various solvents such as water, non-aqueous polar solvents, and nonpolar solvents. In general, the PISA of an amphiphilic block copolymer requires a solvophilic, stabilizing block, which is chain-extended by a soluble or miscible monomer in the polymerization solvent. The chain extension forms a solvophobic block, and once it reaches the critical degree of polymerization (DP), self-assembly occurs in situ. After self-assembly, the remaining monomer becomes encapsulated in freshly formed nanoparticles, and its spatial proximity to the growing chain end accelerates the polymerization. Due to the versatility of this method, PISA has been studied for a wide range of applications from Pickering emulsion stabilization to drug delivery systems. In contrast, the traditional block copolymer PISA remains a two-step process to synthesize the first block, followed by a combined polymerization and assembly of the second block.

Gradient copolymerization-induced self-assembly (gPISA) enables the direct synthesis of amphiphilic copolymer nanoparticles from monomers in a single step. gPISA uses the statistical copolymerization of two monomers with different reactivities. The more reactive monomer is more quickly incorporated into the chain, so it is more represented in the copolymer toward the initiator end.^4−6^ Meanwhile, the incorporation of the second slower-reacting monomer is predominant at the end of polymerization when the first monomer has been depleted. When the two monomers have different solvophilicities, amphiphilic gradient copolymers are formed, which can similarly self-assemble in specific solvents in a similar fashion to block copolymer analogs.^3^ Finally, gradient copolymerization can be performed as PISA (gPISA) if the slower-reacting solvophobic monomer is soluble or miscible with the polymerization solvent to form an insoluble core-forming segment.

Compared with the traditional block copolymer PISA, gPISA is more restricted to the choice of monomers, taking into account not only the monomer/polymer solvophilicity but also the reactivity ratios of both comonomers. Therefore, there are very few examples of gPISA in the literature. Choi and co-workers directly synthesized fractal nanoparticles from monomers using the ring-opening metathesis copolymerization in tetrahydrofuran.^7^ More recently, Zhang and co-workers used the accelerated reactivity of acrylate monomers compared with that of methacrylates in frustrated Lewis pair in gPISA of dimethylaminoethyl acrylate with fluorinated methacrylates in toluene.^8^ Boyer and co-workers used the faster incorporation of methacrylates in copolymerization with acrylamides in the single-step synthesis of nanoparticles based on the statistical copolymerization of oligo(ethylene glycol) methyl ether methacrylate (OEGMA) with diacetone acrylamide in water, which leads to spherical or worm-like micelles.^9^

Fluorine magnetic resonance imaging (^19^F MRI) has emerged as an extensively studied diagnostic method in recent decades.^10^ Compared to conventional clinically used hydrogen (^1^H) MRI, ^19^F MRI benefits from the absence of a fluorine background signal in the body, which enables specific and accurate visualization of the fluorotracer biodistribution.^11^ Among numerous studied fluorinated tracers, semifluorinated polymers stand out as a robust and highly versatile platform for ^19^F MRI.^12−14^ In particular, self-assembled fluorinated copolymer nanoparticles are an exciting platform for theranostics, since they enable the encapsulation of hydrophobic drugs into the micellar core to facilitate simultaneous therapy and polymer nanocarrier tracing.^15,16^

The straightforward synthesis of self-assembled ^19^F MRI tracers using PISA has been reported by several groups. Whittaker and colleagues synthesized block copolymer nanoparticles with fluorine-containing hydrophilic shells by dispersion PISA of styrene in isopropanol.^17^ The nanoparticles were visualized by ^19^F MRI in vitro; however, the fluorine content was relatively low (<3 wt %). Our research group synthesized fluorinated core nanoparticles by dispersion PISA of *N*-(2,2,2-trifluoroethyl)acrylamide in water via a chain extension of a PEG-based macromolecular chain transfer agent (CTA).^18^ The fluorine content in the nanoparticles was relatively high (>20 wt %), but the restricted chain mobility of the fluorinated block in the rigid micelle core significantly affected the relaxation time and attenuated the ^19^F MRI signal. This issue was partially solved by plastifying the fluorinated core with an *N*-(2-hydroxyethyl)acrylamide comonomer.

We hypothesized that the gradient copolymer microstructure would improve the ^19^F MRI signal compared to analogous block copolymer nanoparticles with rigid fluorinated cores due to the (1) fluorines with favorable NMR relaxation properties in the micelle shell and (2) hydrophilic units in the fluorinated core, which would lead to plasticization and extended transverse relaxation times. Furthermore, performing the nanoparticle synthesis in a single step from monomers using gPISA will facilitate the entire synthetic process and make such fluorotracers more accessible.

This work is the first report of a single-step synthesis of amphiphilic fluorinated copolymer nanoparticles directly from monomers via gradient polymerization-induced self-assembly (gPISA) in water. We used different reactivities in the statistical copolymerization of poly(ethylene glycol) methacrylate and *N*-(2,2,2-trifluoroethyl)acrylamide. Both monomers are water-soluble, but during polymerization, a composition gradient is formed, and the nanoparticles are directly formed when the fluorine-rich chain segment reaches the critical length to become hydrophobic. The copolymers and nanoparticles were characterized using size-exclusion chromatography (SEC), NMR, dynamic light scattering (DLS), and transmission electron spectroscopy (TEM). Analogous diblock copolymer nanoparticles were synthesized via traditional PISA to compare the ^19^F MRI performance of the gradient and diblock copolymer micelles. As anticipated, the gradient copolymer nanoparticles outperformed the block copolymers in ^19^F MRI properties, which indicates their potential as next-generation fluorinated nanotracers.

## 2 Experimental Section

### 2.1 Materials

Poly(ethylene glycol) methacrylate (PEGMA) (*M*_n_ = 500 g mol^–1^) (Sigma-Aldrich) was filtered through a short basic alumina column to remove the inhibitor. *N*-(2,2,2-Trifluoroethyl)acrylamide (TFEAM) was synthesized according to the literature protocol.^19^ 2,2′-Azobis[2-(2-imidazolin-2-yl)propane] dihydrochloride (VA-044) purchased from TCI Europe. The rest of the chemicals, counting 1,3,5-trioxane, 4-((((2-carboxyethyl)thio)carbonothioyl)thio)-4-cyanopentanoic acid (CTCPA), 2,2′-azobis(2-methylpropionamidine) dihydrochloride (V-50), *N*,*N*-dimethylformamide (DMF) were purchased from Sigma-Aldrich and used without further purification. Water was deionized with a Millipore Milli-Q water purification system.

### 2.2 Synthesis and Kinetic Studies of Gradient Copolymer PPEGMA_m_-*grad*-TFEAM_n_

Fluorine-containing polymers were synthesized via RAFT-mediated aqueous dispersion gradient copolymerization, a method chosen to control the polymerization process and its ability to produce polymers with controlled structures. In this detailed experimental setup, the inhibitor-free poly(ethylene glycol) methacrylate (PEGMA) (0.05 mmol, 26 mg) and TFEAM (0.21 mmol, 33 mg) monomers, chain transfer agent CTCPA (0.001 mmol, 0.331 mg), VA-044 (0.11 mg, [CTA]_0_/[VA-044]_0_ = 3:1), were weighed and transferred to a polymerization vial (total solids content 6 wt %), along with 1,3,5-trioxane (5 mg) internal standard was dissolved in 0.922 mL of demineralized water. The reaction vail is closed and purged with nitrogen gas for about 10 min. Later, the reaction mixture is stirred and heated to 50 °C in an aluminum heating block. Aliquots were taken under N_2_ for analysis, such as ^1^H NMR, SEC, and dynamic light scattering (DLS) during the reaction at predetermined intervals. The monomer conversions were calculated using ^1^H NMR (DMSO-*d*_6_) relative to the 1,3,5-trioxane internal standard (chemical shift 5.11 ppm). TFEAM conversions were determined by comparing the residual vinyl peaks at 5.7–5.75 ppm. The copolymerization kinetic data have been fitted with the Meyer–Lowry equation to obtain the reactivity ratios for both comonomers, and the copolymer microstructure was predicted using the Skeist model and kinetic Monte Carlo simulation (ESI).^20,21^

### 2.3 Synthesis of PEGMA_50_-CTCPA macroCTA

Water-soluble trithiocarbonate chain end macro chain transfer agent (CTA) was synthesized by aqueous RAFT polymerization of PEGMA using 4-((((2-carboxyethyl)thio)carbonothioyl)thio)-4-cyanopentanoic acid (CTCPA) CTA. In a typical experimental setup, monomer PEGMA (1.48 mmol, 740 mg), CTCPA (0.029 mmol, 9.09 mg), 2,2′-azobis(2-methylpropionamidine) dihydrochloride (V-50) (0.009 mmol, [CTA]_0_/[V-50]_0_ = 3:1) are added to a polymerization vial, dissolved in 3.6 mL water-DMF mixture (10 v/v % of DMF). The reaction mixture is sealed and purged with nitrogen gas for 15 min. The RAFT polymerization was carried at 70 °C for 5 h under constant stirring and polymerization termination by exposing the reaction to air quenching the radicals. ^1^H NMR and SEC were used to analyze the total conversion of the polymerization.

### 2.4 Synthesis of Diblock Amphiphilic Copolymer PEGMA_m_-*b*-PTFEAM_n_

Chain extension of the water-soluble macroCTA is done through aqueous dispersion RAFT polymerization. This experiment comprises PEGMA_50_-CTCPA (0.001 mmol, 48.7 mg), TFEAM (0.07 mmol, 11.2 mg) and VA-044 initiator ([CTA]_0_/[V-50]_0_ = 4:1, 0.11 mg) were dissolved in 0.929 mL of water. Polymerization vial is sealed and purged with nitrogen gas. The reaction is carried at 50 °C in an aluminum heating block for 3 h. The polymerization was quenched by exposure to air, and samples were taken for ^1^H NMR and SEC characterizations.

### 2.5 Characterization of Polymers and Self-Assembled Nanoparticles

#### 2.5.1 Size Exclusion Chromatography

Size exclusion chromatography (SEC) was used to determine the average molar masses (*M*_n_—number-average molar mass, *M*_w_—weight-average molar mass) and dispersity (*D̵* = *M*_w_/*M*_n_) of the polymers using Malvern OMNISEC system, equipped with OMNISEC RESOLVE and OMNISEC REVEAL modules, including an autosampler, a light scattering detector (RALS 90° angle, LALS 7° angle, 640 nm laser), a differential refractive index (RI) detector, a viscometer and a diode-array-based UV/vis spectrometer. The separation was performed on two PLgel 5 μm mixed-D columns in a series thermostated at 55 °C in *N*,*N*-dimethylacetamide (DMAc) containing 50 mM of LiCl at an elution rate of 0.5 mL min^–1^. Molar masses and dispersities were determined against narrow-dispersity poly(methyl methacrylate) standards.

#### 2.5.2 Nuclear Magnetic Resonance

Nuclear magnetic resonance (NMR) spectra were recorded at 25 °C using a Bruker Advance MSL 400 MHz NMR spectrometer in CD_3_OD, DMSO-*d*_6_, or a 90/10 v/v % mixture of H_2_O/D_2_O. Unless specified otherwise, all ^19^F NMR spectra were measured at a concentration of 60 mg mL^–1^ with the following parameters: a pulse width of 20 μs, a relaxation delay of 8 s, an acquisition time of 1.5 s, and 64 scans, with all chemical shifts reported in ppm. The NMR spectra were processed with MestReNova 14.1.1 software, utilizing its built-in function to calculate signal-to-noise ratios (SNRs).

#### 2.5.3 Dynamic Light Scattering

Dynamic light scattering (DLS) measurements were employed to determine the hydrodynamic diameters (*D*_h_) of the polymers in distilled water using a ZEN3600 Zetasizer Nano-ZS zeta potential analyzer (Malvern Instruments, UK). Before measurement, polymer samples (*c*_pol_ = 1 mg mL^–1^) were stirred overnight for equilibration and filtered through a 0.45 μm PTFE syringe filter. The apparent *Z*-averaged hydrodynamic diameter of the particles, *D*_h_, was determined at a scattering angle of θ = 173°, with data evaluation performed using the Zetasizer DTS (Nano) software.

#### 2.5.4 Transmission Electron Microscopy

Transmission electron microscopy (TEM) was performed under a Tecnai G2 Spirit Twin 120 kV TEM (FEI, Czech Republic). The aqueous solutions of the nanoparticles (3 μL, *c*_pol_ = 1 mg mL^–1^) were dropped on a copper TEM grid coated with a thin electron transparent carbon film. Before use, the grids were treated by glow discharge (Expanded Plasma Cleaner; Harrick Plasma, USA) to hydrophilize the carbon surface. After 2 min, the excess solution was removed by touching the bottom of the grids with filtering paper to minimize oversaturation during the drying process. Additionally, the samples were negatively stained with uranyl acetate (2 μL of 1 wt %) solution dropped onto the dried nanoparticles and removed after 30 s as described above. Lastly, the samples were left to dry completely at room temperature and then observed under the TEM in bright-field imaging mode.

#### 2.5.5 Magnetic Resonance Relaxation

Magnetic resonance relaxation properties of the prepared fluorinated gradient copolymers and diblock copolymers were performed using a 1.5T Minispec relaxometer (Bruker Biospin, Germany) with interchangeable radiofrequency (RF) coils tuned to an optimal resonance frequency of 60 MHz for ^1^H and 54 MHz for ^19^F, correspondingly. The samples were examined at a polymer concentration of 60 mg mL^–1^ at 20 and 37 °C, respectively. *T*_1_ relaxation times were measured using a standard inversion recovery sequence, with the following parameters: repetition time, *T*_R_ = 0.01–10.000 ms; recycle delay, RD = 1 s; scans = 2; 10 points per fitting. For *T*_2_ relaxation times, the Carr–Purcell–Meiboom–Gill (CPMG) sequence was employed: echo time, *T*_E_ = 0.04 ms; *T*_R_ = 5000 ms; RD = 2 s; number of scans = 8; 20,000 points per fitting.

#### 2.5.6 The Magnetic Resonance

The magnetic resonance (MR) properties of fluorinated nanoparticle micelles were quantified using a 7 T MR scanner (Bruker Biospec 70/30, Ettlingen, Germany). To characterize an aqueous phantom sample (polymer concentration of 60 mg mL^–1^ at a volume of 400 μL), we selected a 30 mm double tunable ^1^H/^19^F surface RF coil (Bruker, Ettlingen, Germany).

To obtain cross-sectional images of hydrogen (^1^H) using MRI for reference, we used a standard fast spin–echo sequence with the following parameters: *T*_R_ = 1000 ms; *T*_E_ = 36 ms; spatial resolution = 0.078 × 0.078 mm^2^; number of acquisitions, NA = 2; turbo factor = 8; scan time, ST = 1 min 4 s.

The sensitivity of the fluorinated tracers was evaluated through the application of ^19^F MR spectroscopy (^19^F MRS) with the single-pulse sequence (*T*_R_ = 1000 ms; number of averages = 1–600, bandwidth, BW = 40.22 ppm). In addition, ^19^F MR spectroscopic imaging (^19^F MRSI) was used to obtain a set of ^19^F MR images from a 2D slab of voxels with the following parameters: *T*_R_ = 200 ms; *T*_E_ = 1.6 ms; BW = 40.22 ppm; spatial resolution = 0.93 × 0.93 × 10 mm^3^; scan time = 3.5–120 min.

#### 2.5.7 Magnetic Resonance Data Processing and Quantitation

The data processing, quantification, and evaluation of ^19^F MRS and ^19^F MRSI were primarily carried out using custom-written scripts in the MATLAB programming environment (Matlab R2023b, The MathWorks, Inc., USA). We evaluated the sensitivity of fluorinated micelles using nonlocalized spectroscopy by calculating their signal-to-noise ratio (SNR_MRS_) as the ratio of the signal amplitude (*S*_*a*_) and amplitude of the noise (*N*_*a*_) (eq 1).
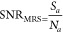
1

Hotspot ^19^F MRSI phantom images were reconstructed as an average of 10 slices from the corresponding resonance frequency range. Voxel values were further normalized for better visual representation. The images were also converted from a 64 × 64 to a 256 × 256 matrix using bilinear interpolation, which allowed us to align them with the dimensions of the reference ^1^H MRI. The mean signal (*S*) within a region of interest (ROI) was divided by the average standard deviation of noise at the corners of the image (σ) to calculate the signal-to-noise ratio (SNR) for ^19^F MRSI images (SNR_MRSI_). We adjusted the SNR calculation by the factor of 0.655 to account for the intrinsic Rician noise distribution in MR images (eq 2).^22^
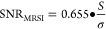
2

### 2.6 Cell Viability Study

The in vitro cytotoxicity of gradient copolymer micelles G2 and G3 was studied using the Panc-1 cell line. Cells were cultured in Dulbecco’s Modified Eagle’s Medium DMEM (Gibco, Thermo Fisher Scientific, USA), supplemented with 10% fetal bovine serum (Gibco, Thermo Fisher Scientific, USA) and 1% Penicillin/Streptomycin (Biosera, France) and incubated under standard conditions (*T* = 37 °C; 5% CO_2_). Initially, Panc-1 cells were seeded in a 96-well plate at a density of 0.1 × 10^6^ cells per well and incubated in a medium for 24 h. Subsequently, cells were treated with various concentrations of G2 respectively G3 nanoparticles (*c*_pol_ = 10, 20, and 30 mg mL^–1^) for 48 h in the cell cultivation medium.

The viability of labeled cells was assessed using the Alamar blue assay (Sigma-Aldrich, St. Louis, MO, USA). Prior to spectrophotometric analysis, cells were washed three times with Dulbecco’s phosphate-buffered saline (DPBS, Gibco, Thermo Fisher Scientific, USA). Then, a 10% Alamar blue solution in cultivation media was added, and cells were incubated under standard conditions for 4 h. Finally, absorbance was measured at wavelengths of 570 and 600 nm using a Tecan Infinite M200 Pro, and the signal from treated cells was compared with that of negative controls. Each experiment was performed in triplicate.

## 3 Results and Discussion

Our previous experiments demonstrated the synthesis of ^19^F MRI block copolymer nanotracers via aqueous dispersion polymerization-induced self-assembly (PISA) of *N*,*N*-(2,2,2 trifluoroethyl)acrylamide (TFEAM) as a novel PISA core-forming fluoromonomer.^18^ The relatively high aqueous solubility of the TFEAM monomer and side chain group with three magnetically equivalent ^19^F nuclei, which provides a singlet ^19^F NMR peak, were key benefits in selecting TFEAM as the PISA core-forming monomer. Inspired by Boyer et al.,^9^ we used the slower incorporation of acrylamides in statistical copolymerization with methacrylates for the single-step gradient copolymerization-induced self-assembly (gPISA). We used acrylamide-based TFEAM as a slower core-forming monomer and methacrylate-based poly(ethylene glycol) methyl ether methacrylate (PEGMA, *M*_n_ = 500 g mol^–1^) as a faster water-soluble shell-forming comonomer.

**Figure 1:**
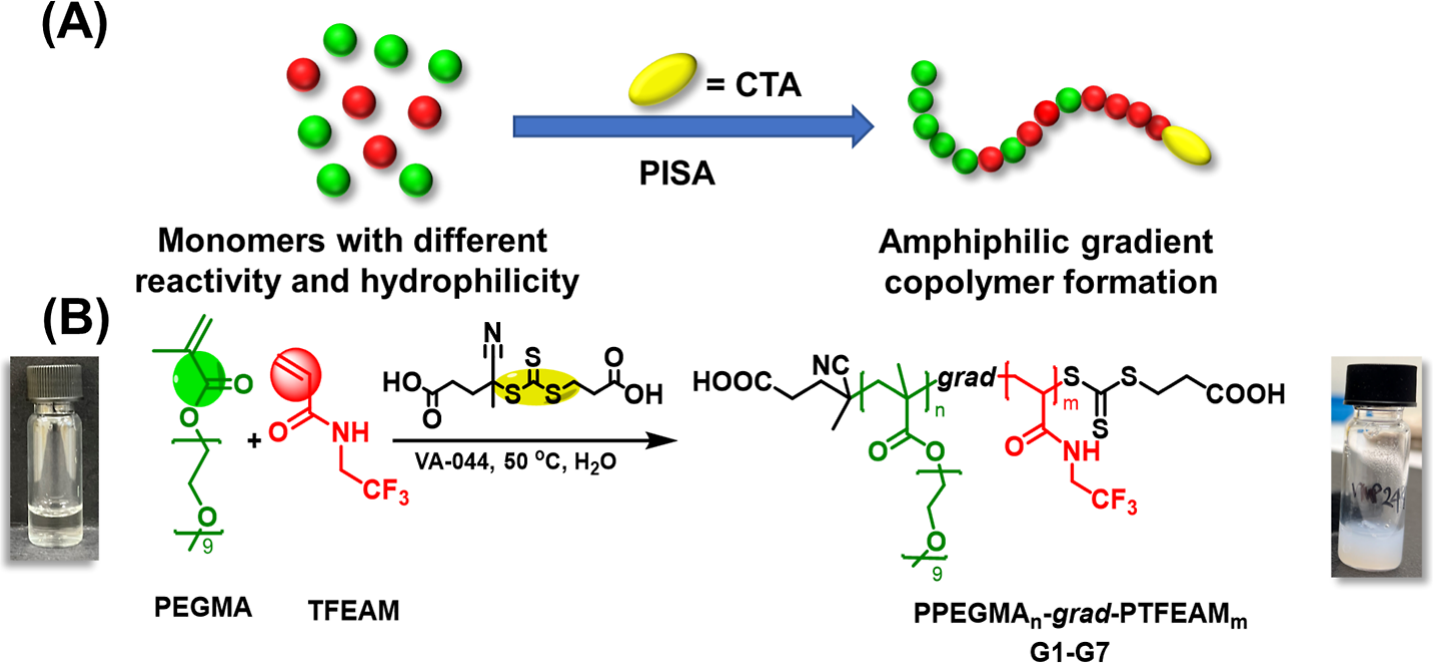
Synthesis of the gradient aqueous RAFT polymerization-induced self-assembly: Schematic illustration of the (A) gradient copolymerization and (B) copolymerization reaction scheme.

Aqueous statistical copolymerizations of PEGMA with TFEAM have been performed via the reversible addition–fragmentation chain transfer (RAFT) technique using a water-soluble 4-((((2-carboxyethyl)thio)carbonothioyl)thio)-4-cyanopentanoic acid (CTCPA) chain transfer agent and VA-044 as a water-soluble azo-initiator (Figure 1).^23^ Polymerizations were performed in distilled water using a constant total solids concentration of 6 wt %, which is below the solubility threshold of TFEAM (∼8 wt %). Using a relatively low monomer concentration helps us prevent the formation of higher-order morphologies during gPISA, as we aim for spherical core–shell micelle tracers. Polymerizations were performed at 50 °C, since higher reaction temperatures often lead to macroscopic precipitation.^2,24^

**Figure 2:**
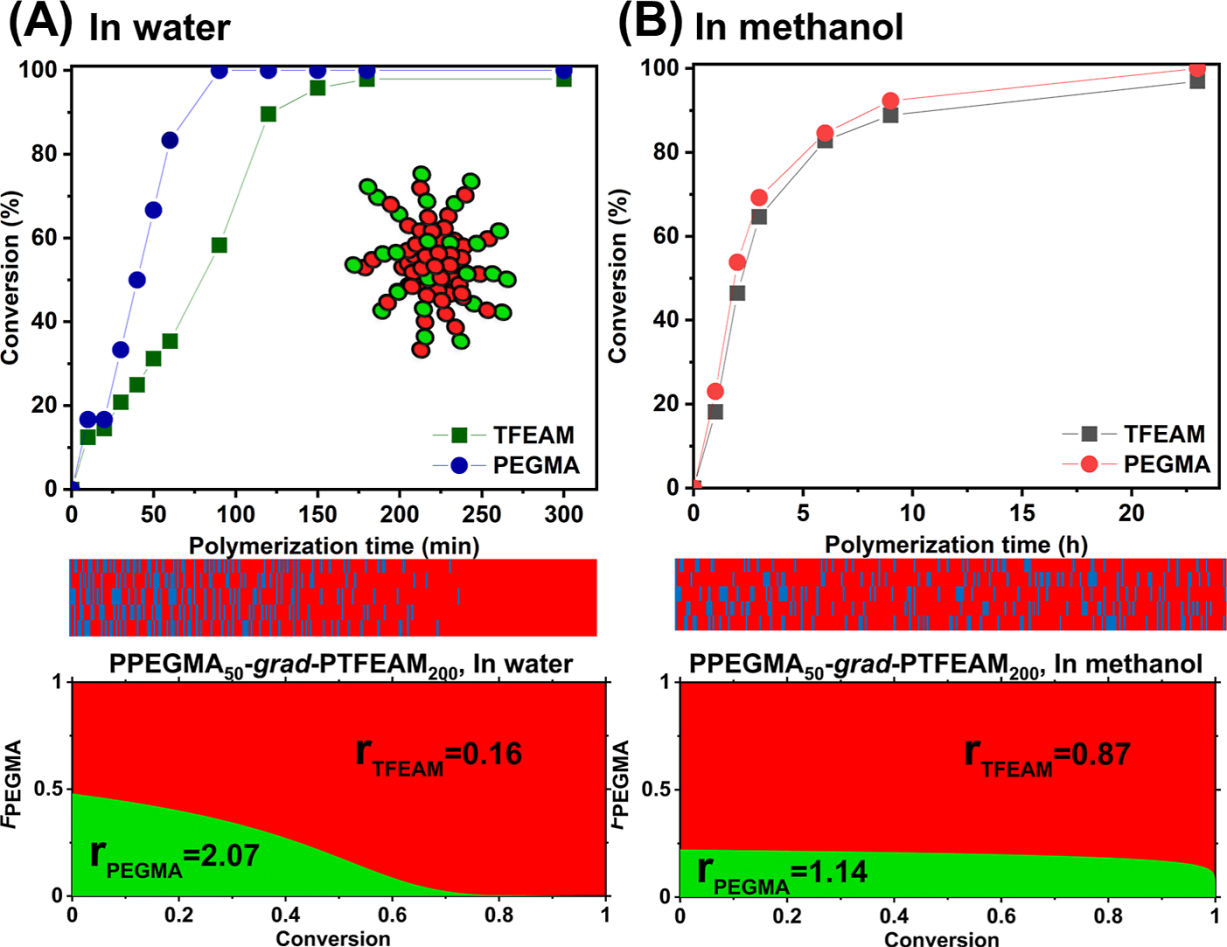
Statistical copolymerization kinetic plots (top), kinetic Monte Carlo simulation of the repeating unit distribution (middle), and Skeist plot (bottom) for gPISA of PEGMA with TFEAM ([PEGMA]_0_/[TFEAM]_0_/[CTCPA]_0_ = 50:200:1) at 50 °C in water (A) and in methanol (B).

First, we studied the statistical copolymerization kinetics of PEGMA and TFEAM in water using two different comonomer ratios [PEGMA]/[TFEAM]/[CTCPA] = 50:200:1 (molar fraction of PEGMA in the comonomer feed, *f*_PEGMA_ = 0.20, Figure 2A) and [PEGMA]/[TFEAM]/[CTCPA] = 50:170:1 (*f*_PEGMA_ = 0.23, Figure S1). In both polymerizations, PEGMA was consumed significantly faster than the TFEAM monomer, which is consistent with the generally described faster copolymerization kinetics of methacrylates compared to acrylamides.^9^ Both statistical copolymerizations yielded amphiphilic gradient copolymers that self-assembled into nanoparticles during gPISA. Due to the formation of nanoparticles, the polymerization of PTFEAM accelerated after reaching the critical degree of polymerization and in situ self-assembly, where the PEGMA-rich chain segment served as a stabilizing part, and the TFEAM-rich chain segment formed a core in the resulting polymer nanoparticle. These trends are consistent with the current understanding of the PISA mechanism, since the self-assembly depends on the (a) critical DP of the core-forming monomer, (b) interactions between chains of the shell, and (c) thermodynamic and kinetic stability competition between the core-forming block and the solvent.^25,26^

Copolymerization parameters for the PEGMA/TFEAM comonomer pair were obtained via an integral model using the Meyer–Lowry equation fitting of copolymerization kinetic data.^20,27^ For the *f*_PEGMA_ = 0.20 copolymerization, the reactivity ratios of the individual monomers were *r*_PEGMA_ = 2.07 and *r*_TFEAM_ = 0.16, which indicates a gradient copolymer architecture. A similar gradient characteristic was observed for the *f*_PEGMA_ = 0.23 copolymer. The compositional distribution along the polymer chain was simulated for an *f*_PEGMA_ = 0.20 monomer feed ratio using the Skeist model.^21^ This result illustrates that the beginning of the copolymer chain is abundant in PEGMA, whereas close to the end of the chain, fluorine-rich PTFEAM prevails, and the last approximately 20% of the chain is a homopolymer of TFEAM. Furthermore, the statistical distribution of individual repeating units in macromolecular chains is modeled via the kinetic Monte Carlo (kMC) simulation (Figure 2A), which plays a crucial role in explaining the high ^19^F MRI intensity of the resulting fluorinated gradient copolymers.^28,29^

Interestingly, the statistical copolymerization of PEGMA with TFEAM is highly solvent-dependent. The difference in reactivity ratios of individual monomers (*r*_PEGMA_ = 1.14, *r*_TFEAM_ = 0.87, Figure 2B) was significantly lower for the gradient copolymerization in methanol, which is a good solvent for both repeating units, than in water. The copolymerization in methanol did not form nanoparticles, and the final copolymer showed a nearly random distribution of repeating units. The solvent-dependent gradient steepness in free radical statistical copolymerizations of methacrylates and acrylamides has been reported in the literature but not to this extent.^30,31^ This result can be explained by the increased solvation of the transition state of the propagation step of hydrophilic CTA with the hydrophilic PEGMA monomer in water, which enabled PEGMA to be preferentially added to the growing chain.

**Figure 3:**
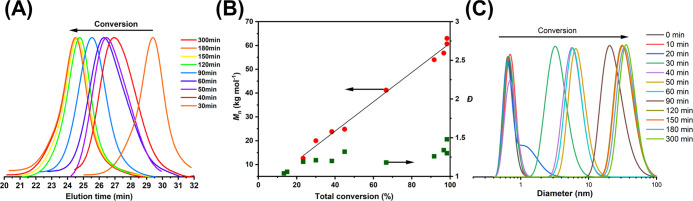
Kinetic study for gPISA of PEGMA with TFEAM ([PEGMA]_0_/[TFEAM]_0_/[CTCPA]_0_ = 50:200:1) at 50 °C in water: (A) SEC trace evolution with copolymerization time. (B) Evolution of the average molecular weight with total monomer conversion. The black line is present to guide the eyes. (C) Evolution of the nanoparticle hydrodynamic size with respect to the copolymerization time using DLS.

**Figure 4:**
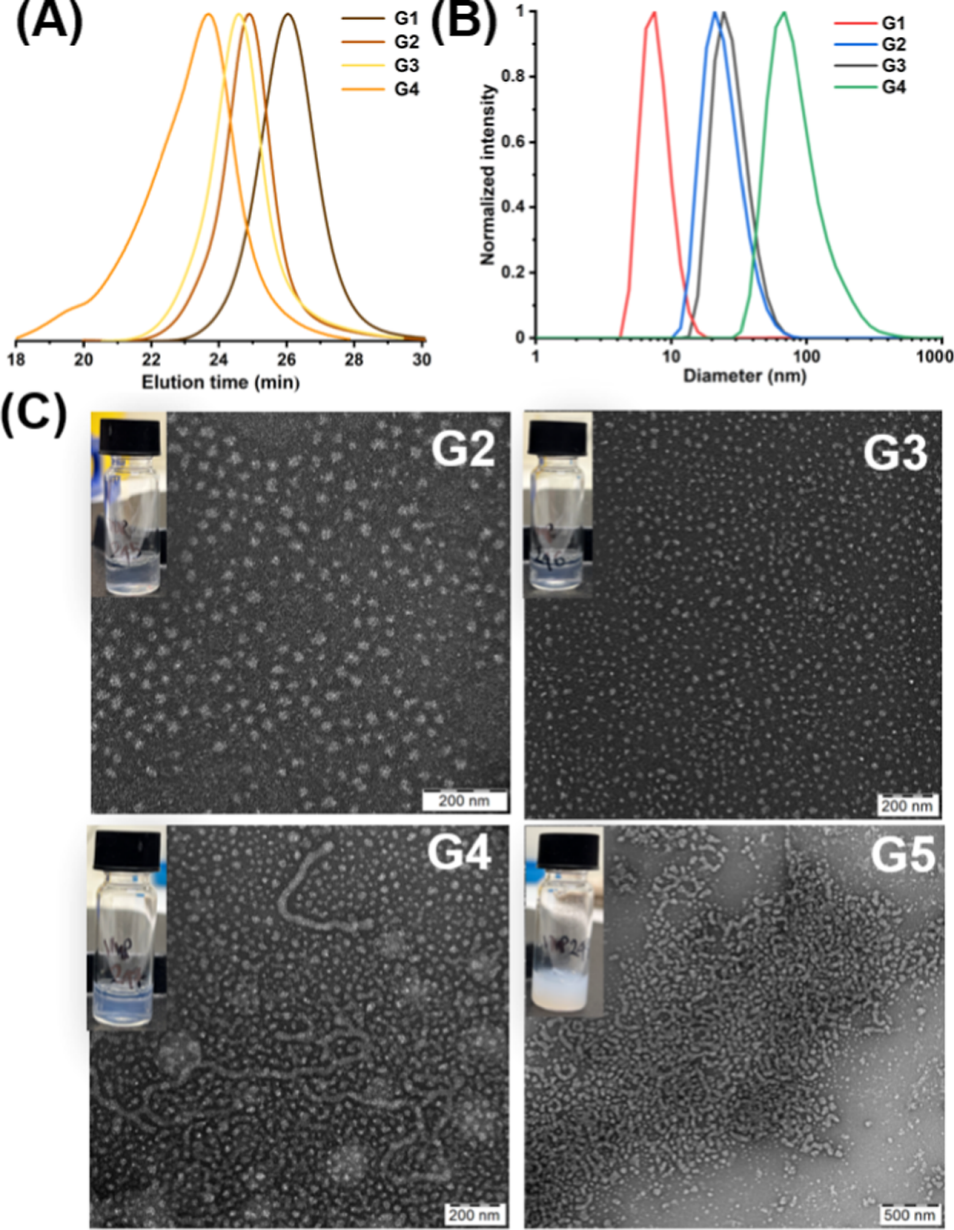
Characterization of the PPEGMA_50_-*grad*-PTFEAM_*x*_ gradient copolymers with different total DP_tot_ = 200–350 (G2–G5): (A) overlay of SEC traces; (B) hydrodynamic diameter distributions of the nanoparticles determined using DLS; and (C) TEM micrographs of the gradient copolymer nanoparticles.

The aqueous gPISA of PEGMA with TFEAM was relatively well controlled, as demonstrated by the increase in molecular weight with the total conversion and low dispersity (Figure 3B). We also monitored the molecular weight distributions using SEC; higher molecular weights were obtained with increasing monomer conversions (Figure 3A). Additionally, the in situ self-assembled copolymer nanoparticles were monitored using DLS. From the plot, we can identify the point at which the growing polymer chains self-assembled to form nanoparticles (Figure 4C).

**Figure 5:**
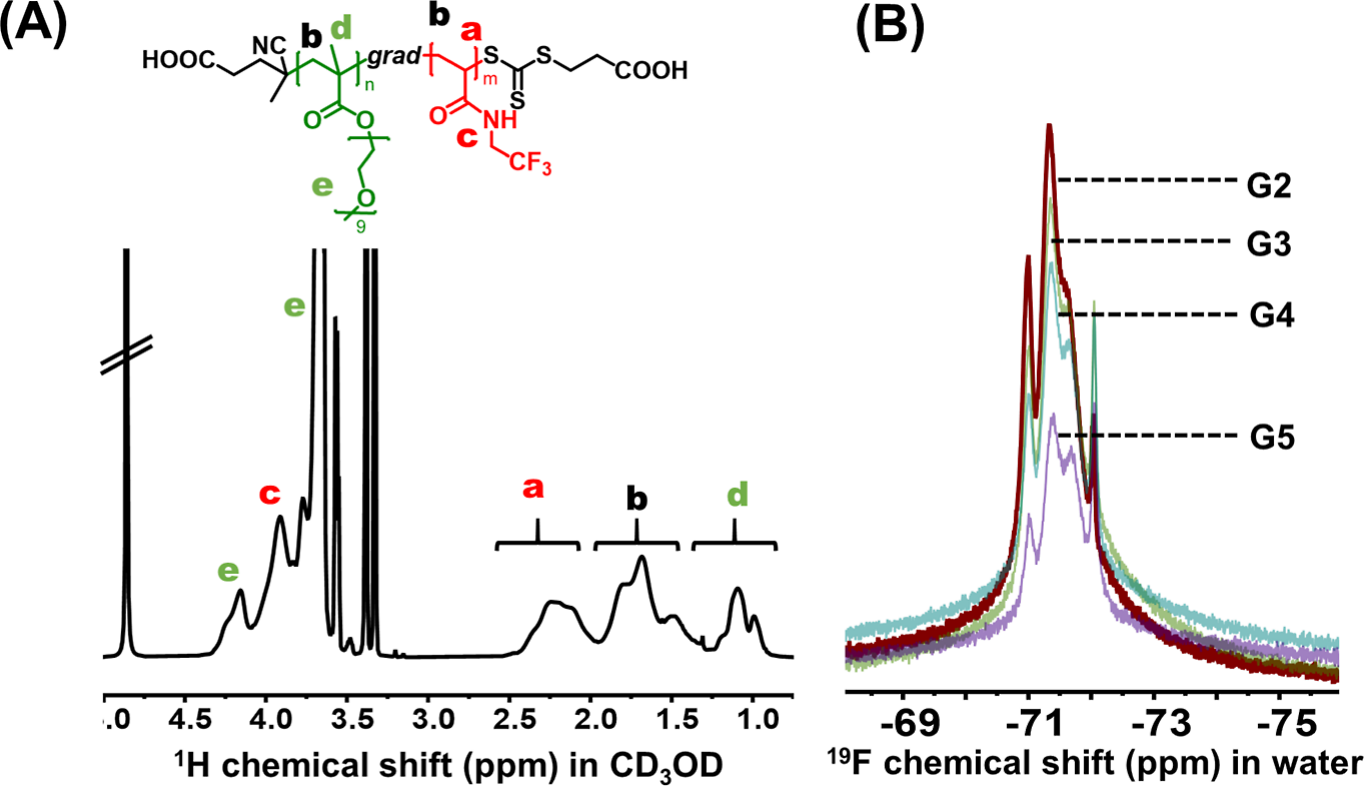
Characterization of the PPEGMA_50_-*grad*-PTFEAM_200_ gradient copolymer (G2); (A) ^1^H NMR spectrum in CD_3_OD; (B) ^19^F NMR spectra of a series of gradient copolymers (G2–G5) in water.

A series of gradient copolymer nanoparticles was synthesized with different comonomer ratios (Table 1) to determine the maximum extent of fluorination that still provides well-stabilized nanoparticles. While the target DP of PEGMA remained constant at DP_PEGMA_ = 50, the target DP of TFEAM systematically varied in the range of 100–300 (beyond this DP, the polymer nanoparticles precipitated from the solution), which corresponded to a fluorine content of 8.6–24 wt %. The total monomer conversion for all compositions was >97%, and the polymer dispersities remained ≤1.2, except at a higher D*P*_tot_ = 350 (Figure 4A). The copolymer composition was determined using ^1^H NMR and corresponded to the monomer feed ratio (Figure 5A), where the overlapping peaks were resolved by 2D ^1^H–^13^C HSQC NMR experiments (Figure S2). The size of the resulting nanoparticles was studied using DLS (Figure 4B), and the morphologies were examined using TEM. The increased hydrophobicity fused the micelle cores and paved the path for the transition from spherical micelles to worm-like micelles (as seen from TEM micrographs). This trend can be observed when the total DPs varied from 100 to 350.^32^ To assess the reproducibility and robustness of our protocol, copolymers G2 and G3 were synthesized in three independent batches, with very good batch-to-batch reproducibility of key properties (Table S1, Figure S3).

**Table 1:** Characteristics of the PPEGMA-*grad*-PTFEAM Gradient Copolymers and Their Nanoparticles Synthesized by the Aqueous Dispersion gPISA^a^

pol.	DP_PEGMA_^b^/DP_TFEAM_	conv. (%)^c^	*f*_PEGMA_^d^	*M*_n_^Theo,^^c^ (kg mol^–1^)	*M*_n_^SEC,^^e^ (kg mol^–1^)	*D̵*^e^	*D*_h_ (nm)/PDI^f^	^19^F NMR SNR^g^
G1	50/50	>99	0.50	32.6	33.1	1.17	7/0.559	n.d.
G2	50/150	>99	0.25	48.2	56.4	1.18	38/0.126	128
G3	50/200	>99	0.20	55.9	62.3	1.22	44/0.080	110
G4	50/250	>99	0.17	63.2	71.6	1.28	76/0.088	92
G5	50/300	>98	0.14	70.9	100.9	2.04	97/0.895	62
G6	100/300	>95	0.25	96.2	78.9	1.33	47/0.090	137
G7	100/400	>95	0.20	111.5	97.6	1.35	61/0.112	106

a All experiments were performed at 50 °C in water at a total solids content of 6 w/w % and [CTCPA]_0_/[VA-044]_0_ = 3.

b Target DPs defined by the initial molar ratio of monomer to CTA.

c Determined by ^1^H NMR.

d Initial molar fraction of PEGMA.

e Determined by SEC against PMMA calibration.

f Determined by DLS in water at *c*_pol_ = 1 mg mL^–1^.

g Determined by 400 MHz ^19^F NMR in water/D_2_O (90/10%) at *c*_pol_ = 60 mg mL^–1^. n.d.—not determined.

The potential of synthesized nanoparticles as ^19^F MRI tracers was first investigated by ^19^F NMR in water. All gradient copolymer nanoparticles presented a broad NMR peak that corresponded to the –CF_3_ side chain groups (Figure 5B). The broad peak shows a multimodal distribution due to the presence of ^19^F nuclei with different surroundings, which originated from a position in the nanoparticle (i.e., fluorines in the shell or the core) or the chemical structure of neighboring repeating units (i.e., the presence of homodyads or heterodyads). Considering the gradient composition and relatively high content of TFEAM in the hydrophilic shell (as demonstrated by the kinetic Monte Carlo simulations), one can anticipate that these shell-originated fluorines may significantly contribute to the relatively high ^19^F NMR signal-to-noise ratios (SNRs) of gradient copolymers.

To demonstrate that the ^19^F NMR signal originates from micelles rather than free unimers, we conducted dilution experiments (Figure S4). The signal intensity decreased linearly with decreasing polymer concentration, passing through the origin. Since the concentration of unimers remains constant above the critical micelle concentration (CMC), a significant change in signal upon dilution indicates that the signal arises from micelles. Thus, the linear decrease confirms that the observed ^19^F NMR signal is predominantly due to micelles. Interestingly, increasing the TFEAM (and thereby fluorine) content resulted in a decrease in ^19^F NMR SNR. This relationship can be explained by the presence of long fluorine-rich chain-end segments, which self-assemble into a rigid micelle core without sufficient segmental mobility and magnetic relaxation to contribute to the ^19^F NMR signal. This has been confirmed by measurement of the ^19^F NMR peak integrals of G2-G5 (Table S2), which dropped with increasing fluorine content, suggesting decreased magnetic “visibility” of the rigid core segments. Furthermore, the neighboring fluorinated repeating units can show strong dipolar interactions that attenuate the ^19^F MR signal.^33^

**Figure 6:**
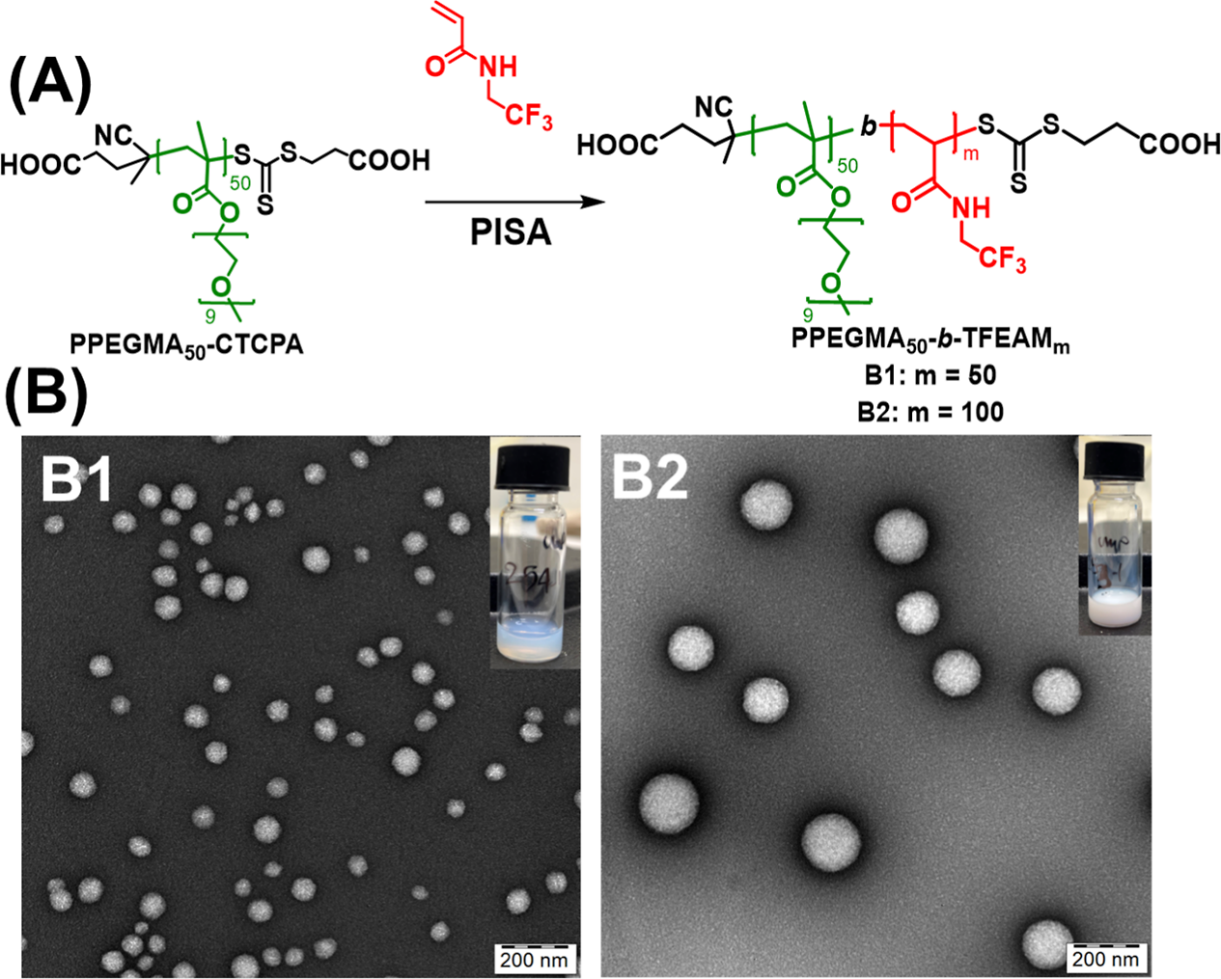
Diblock copolymer nanoparticles of PPEGMA_50_-*b*-PTFEAM_*x*_ (*x* = 50, 100) synthesized by aqueous dispersion PISA. (A) Reaction scheme and (B) TEM images of spherical nanoparticles.

The gradient copolymer nanoparticles were compared with analogous block copolymers synthesized by the traditional two-step PISA protocol. First, a P(PEGMA)_50_ bottlebrush macroCTA was synthesized via an aqueous RAFT protocol with CTCPA CTA in a water-DMF mixture. The complete conversion of polymerization was confirmed by ^1^H NMR, and the purified polymer was characterized by SEC (*D̵* = 1.01, *M*_n_ = 23 kDa). In the second step, this PPEGMA macroCTA was used in the aqueous dispersion PISA of the TFEAM monomer with the target DP_TFEAM_ of 50 and 100. Interestingly, the diblock copolymer nanoparticles were colloidally unstable when DP_TFEAM_ was greater than 100. The spherical morphology of the nanoparticles was visualized via TEM. The diblock polymeric micelles showed a more defined spherical structure, which implies the presence of a more rigid fluorinated core (Figure 6). As we hypothesized, the ^19^F NMR signal intensities of the diblock copolymer nanoparticles in a water–D_2_O (9:1) mixture were significantly lower than the SNRs of the gradient copolymer nanotracers synthesized by gPISA.

**Figure 7:**
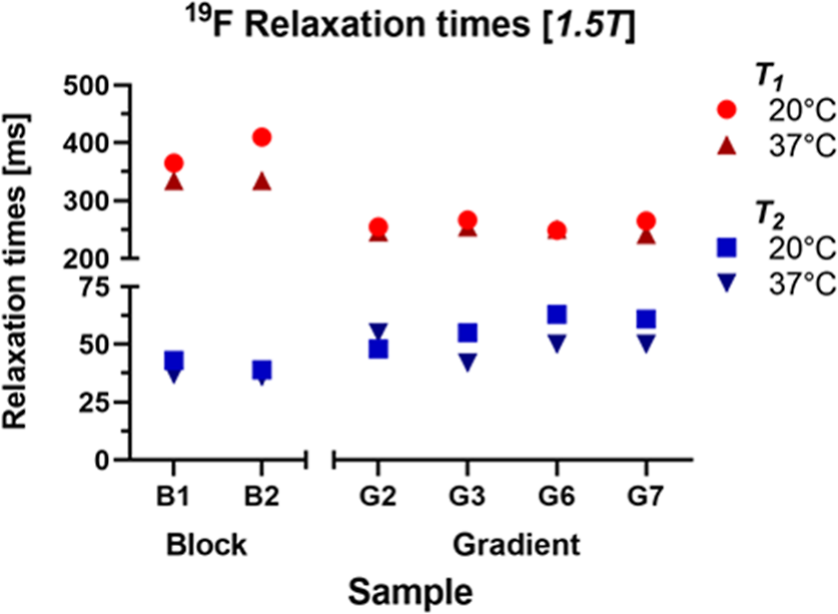
^19^F MR relaxation times of the PPEGMA-*co*-PTFEAM gradient and respective block copolymer nanoparticles in water.

The magnetic relaxation times are key parameters that influence the diagnostic potential of ^19^F MRI nanotracers. The spin–lattice relaxation time (*T*_1_) should be low, whereas the spin–spin relaxation time (*T*_2_), which is related to the fluorinated chain mobility, should be as high as possible to ensure a high MR signal and consequently good sensitivity of the tracer.^11,34^ Herein, MR relaxometry (1.5T) was used to study the ^19^F MR relaxations of the optimized gradient and block copolymer nanoparticles prepared by gPISA and traditional PISA (Figure 7). These measurements indicate gradient copolymers had better *T*_1_ values (*T*_1_ = 250–300 ms) than block copolymers (*T*_1_ = 350–450 ms). More importantly, gradient copolymers had longer *T*_2_ (*T*_2_ > 50 ms) than block copolymer nanoparticles (*T*_2_ < 40 ms). Similar relaxation behavior was recently observed in ^31^P MRI studies of amphiphilic gradient-type polyphosphonate copolymers.^35^ This property, the higher fluorine content, and the presence of magnetically well “visible” fluorines in the hydrophilic micelle shell contributed to the superior ^19^F MRI properties of gradient copolymer nanotracers.

**Figure 8:**
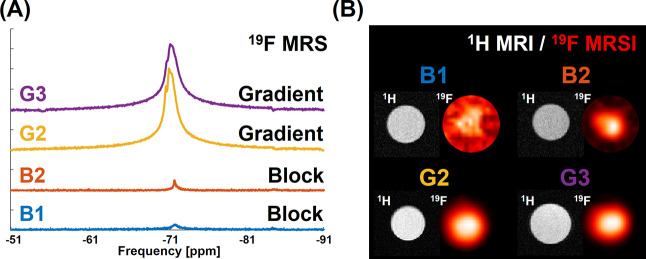
^19^F MRS and MRSI for gradient and block copolymer nanoparticles in water (*c*_pol_ = 60 mg mL^–1^) (A) ^19^F MRS (scan time 10 min); (B) ^1^H MRI and ^19^F MRSI (scan time 2 h).

Finally, the ^19^F MRI potential of synthesized nanotracers was demonstrated by the in vitro ^19^F MR spectroscopic imaging (MRSI) of tracers in Eppendorf tube phantoms using a preclinical 7T MRI instrument. Both fluorinated nanotracer classes (gradient and block copolymers) were successfully visualized in vitro. However, the gradient copolymer tracers had significantly greater imaging intensities than the block copolymers at the same polymer concentration, where the signal intensity remained lower (SNR <10) after a 120 min acquisition (Figures 8 and S5–S7). The higher ^19^F MRI performance of gradient nanoparticles can be ascribed to the presence of TFEAM units and plasticization of the fluorinated core by the hydrophilic comonomer. A similar behavior was recently observed for non-PISA synthesized fluorinated gradient copoly(2-oxazoline)s.^36^ On the other hand, the ^19^F MRI sensitivity of our tracers was slightly lower than the sensitivity observed for PFCE nanoemulsions, which is, however, compensated by increased nanoformulation stability and more straightforward synthesis of our systems.^37^ Based on the combination of the ^19^F MRI performance, micelle size, and spherical morphology, gradient copolymer nanoparticles G2 and G3 were selected for further investigation.

The cytocompatibility of the as-synthesized nanotracers G2 and G3 was confirmed by the Alamar blue assay in the human pancreatic cancer cell line Panc-1 (Figure S8). The cell viability was evaluated after 48 h of incubation with nanoparticles at three different concentrations (*c*_pol_ = 10, 20, and 30 mg mL^–1^). Neither nanotracers presented any apparent cytotoxicity at a concentration of 10 mg mL^–1^, which demonstrates their good cytocompatibility, particularly for intravenous applications when the polymer is rapidly diluted in a blood pool. At 20 mg mL^–1^, the G2 nanoparticles remained nontoxic; however, longer G3 nanoparticles were cytotoxic, presumably due to the higher content of hydrophobic PTFEAM units. Finally, the nanoparticles at the highest tested concentration were cytotoxic for both tracers. To conclude, the nanoparticles exhibited very good cytocompatibility (particularly the G2 tracer), which is a necessary prerequisite for in vivo MRI tracing experiments. However, these in vivo experiments are beyond the scope of this study, since this study focused on the straightforward synthesis of nanotracers by gPISA and their in vitro MRI properties. In vivo imaging experiments are in progress and will be reported in the near future.

## 4 Conclusions

In summary, we developed a one-step method to synthesize fluorinated polymer nanoparticles from monomers via aqueous gradient copolymerization-induced self-assembly (gPISA). In this process, we used monomers of different reactivities and hydrophilicities: poly(ethylene glycol) methacrylate (PEGMA) and *N*,*N*-(2,2,2-trifluoroethyl)acrylamide (TFEAM). Our results demonstrate that compared with TFEAM, PEGMA is preferentially incorporated into the growing polymeric chain because of its higher reactivity (*r*_PEGMA_ = 2.07, *r*_TFEAM_ = 0.16). The core-forming TFEAM monomer is water-soluble but becomes insoluble upon polymerization. The gradient copolymerization directly forms nanoparticles, where PEGMA acts as a stabilizing shell, and the PTFEAM-rich segment induces the in situ self-assembly. The prepared nanoparticles have a spherical or worm-like morphology depending on the comonomer ratio.

Compared with analogous block copolymers, the synthesized gradient copolymer nanoparticles exhibit superior ^19^F MRI performance in vitro due to the improved fluorine relativity, which presumably results from the well-relaxing TFEAM repeating units in the micelle shell and plasticization of the fluorinated core by the hydrophilic monomer. The noncytotoxic characteristic of gradient nanoparticles was demonstrated in Panc-1 cells. Given their straightforward synthesis and superior imaging capabilities, these gradient copolymer nanoparticles hold great promise for advancing diagnostic technologies and potentially will improve the sensitivity and specificity of future medical diagnostics. Finally, we recognize the concerns surrounding fluorinated compounds due to environmental regulations on perfluoroalkyl substances. However, the specialty fluorinated polymers developed here are designed for use in minimal quantities, specifically for high-sensitivity medical imaging, which offers significant health benefits. Unlike bulk-use perfluoroalkyl substances, these polymers contribute to life-saving diagnostics with very low environmental impact. We believe that the medical benefits justify their use, even amid potential regulatory changes.

## Supporting Information Available

Copolymerization kinetic plots, additional characterization of copolymers and nanoparticles by SEC, NMR, DLS and ^19^F MRI, cytotoxicity data (PDF)
