# Supplementary material for: Single-Step Synthesis of Highly Sensitive 19F MRI Tracers by Gradient Copolymerization-Induced Self-Assembly
Source: Biomacromolecules. 2024 Nov 19;25(12):7685–94. doi: 10.1021/acs.biomac.4c00915 (PMC11632659; doi:10.1021/acs.biomac.4c00915)
Supplement: Supplementary file 1 — bm4c00915_si_001.pdf [file bm4c00915_si_001.pdf]

Supporting Information

Single-Step Synthesis of Highly Sensitive ^{19}F MRI Tracers by Gradient Copolymerization-Induced Self-Assembly

Vyshakh M. Panakkal,[a] Dominik Havlicek,[b,d] Ewa Pavlova,[c] Klara Jirakova,[b,e] Daniel Jirak[b,d,f] and Ondrej Sedlacek,[a,*]

[a]Department of Physical and Macromolecular Chemistry, Faculty of Science, Charles University, 128 40 Prague 2, Czech Republic

[b]Department of Diagnostic and Interventional Radiology, Institute for Clinical and Experimental Medicine, Videnska 1958/9, 140 21 Prague, Czech Republic

[c]Institute of Macromolecular Chemistry, v.v.i., Academy of Sciences of the Czech Republic, Heyrovsky Sq. 2, 162 06 Prague 6, Czech Republic

[d]Institute of Biophysics and Informatics, First Faculty of Medicine, Charles University, Kateřinská 1660/32, 121 08 Prague, Czech Republic

[e]Third Faculty of Medicine, Charles University, Ruská 87, 100 00 Prague, Czech Republic

[f]Faculty of Health Studies, Technical University of Liberec, Studentská 1402/2, 46117 Liberec, Czech Republic

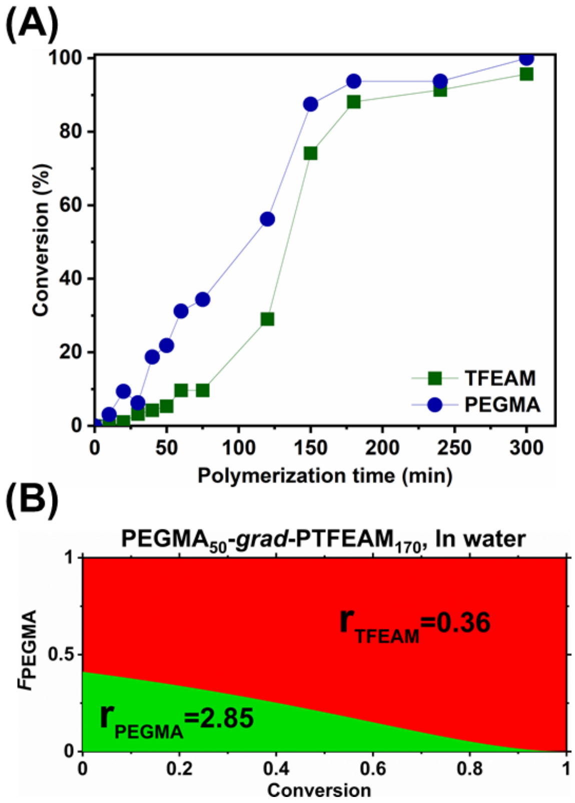

Figure S1. Kinetic plots for the gPISA synthesis of $PPEGMA_{50}$-*grad*-$PTFEAM_{170}$ (A) Conversion of the monomers with polymerization time (B) Skeist plot.

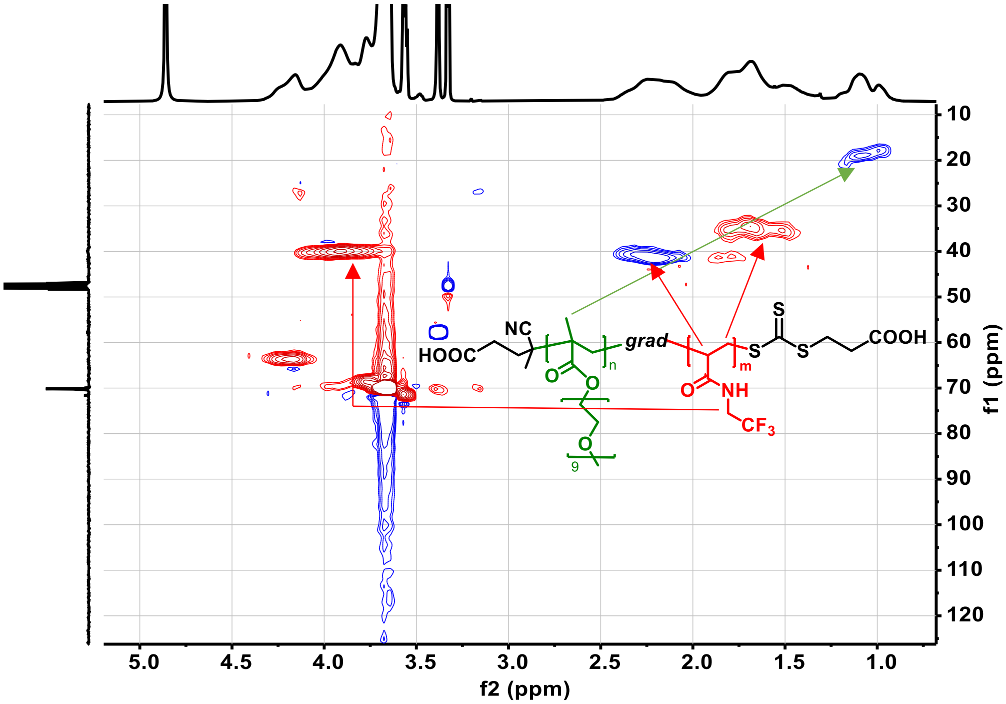

Figure S2. 2D HSQC NMR of the gradient copolymer G2 in CD_3OD.

Table S1. Characteristics of the PPEGMA-*grad*-PTFEAM gradient copolymers and their nanoparticles synthesized by the aqueous dispersion gPISA. Samples G2 and G3 were prepared in three independed batches

Pol.	DP_{PEGMA}[b] /DP_{TFEAM}	Conv. (%)[c]	f_{PEGMA}[d]	$M_n^{Theo,c}$ (kg mol^{-1})	$M_n^{SEC,e}$ (kg mol^{-1})	$Đ$[e]	D_h(nm) /PDI[f]	^{19}F NMR SNR[g]
G1	50/50	>99	0.50	32.6	33.1	1.17	7/0.559	n.d.
G2	50/150	>99	0.25	48.2	56.4	1.18	38/0.126	128
G2.a	50/150	87	0.25	41.7	39.1	1.14	32/0.968	87[h]
G2.b	50/150	96	0.25	46.0	39.7	1.13	30/0.590	87[h]
G2.c	50/150	97	0.25	46.5	38.5	1.18	30/0.846	84[h]
G3	50/200	>99	0.20	55.9	62.3	1.22	44/0.080	110
G3.a	50/200	94	0.20	52.2	45.0	1.22	41/0.340	67[h]
G3.b	50/200	96	0.20	53.3	47.9	1.18	42/0.040	60[h]
G3.c	50/200	97	0.20	53.9	44.9	1.18	35/0.366	70[h]
G4	50/250	>99	0.17	63.2	71.6	1.28	76/0.088	92
G5	50/300	>98	0.14	70.9	100.9	2.04	97/0.895	62
G6	100/300	>95	0.25	96.2	78.9	1.33	47/0.090	137
G7	100/400	>95	0.20	111.5	97.6	1.35	61/0.112	106

[a]All experiments were performed at 50°C in water at a total solids content of 6 w/w% and $[CTCPA]_0/[VA\text{-}044]_0 = 3$. [b]Target DPs defined by the initial molar ratio of monomer to CTA. [c]Determined by ^{1}H NMR. [d]Initial molar fraction of PEGMA. [e]Determined by SEC against PMMA calibration. [f]Determined by DLS in water at c_{pol} = 1 mg mL^{-1}. [g]Determined by Bruker 400 MHz ^{19}F NMR in water/D_2O (90/10%) at c_{pol} = 60 mg mL^{-1}. [h]Determined using 400 MHz Bruker Neo instrument. n.d. - not determined

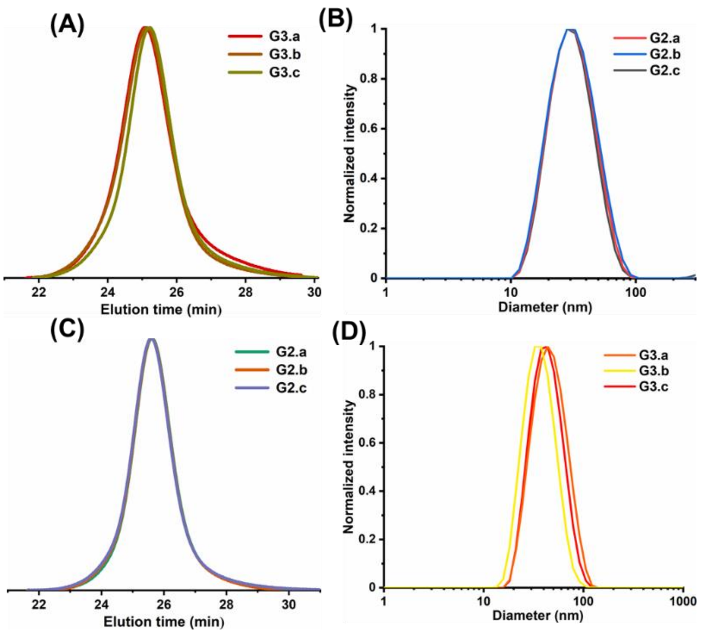

Figure S3. Characterization of the PPEGMA$_{50}$-*grad*-PTFEAM$_n$ (n =150,200) gradient copolymers (A) SEC chromatograms of PPEGMA$_{50}$-*grad*-PTFEAM$_{150}$ (B) DLS plots for the polymer nanoparticle PPEGMA$_{50}$-*grad*-PTFEAM$_{200}$ (c_{pol} = 1 mg/ml, in water) (C) SEC chromatograms of PPEGMA$_{50}$-*grad*-PTFEAM$_{200}$ (D) DLS plots for the polymer nanoparticle PPEGMA$_{50}$-*grad*-PTFEAM$_{150}$ (c_{pol} = 1 mg mL^{-1}, in water).

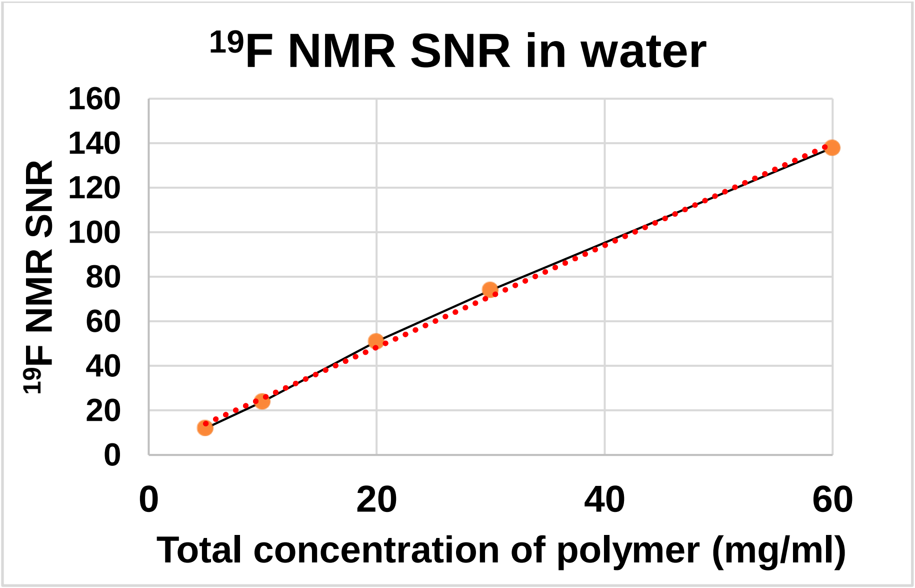

Figure S4. Concentration dependence of ^{19}F NMR SNR of gradient copolymer nanoparticles G2 in water

Table S2. ^{19}F NMR quantification of the fluorine in nanoparticles synthesized by the aqueous dispersion gPISA at constant polymer concentration (c_{pol} = xx mg mL^{-1})

Sample	F count (wt.%)[a]	Relative integral[b]
G2/MeOD	17.83	100
G2/D_2O	17.83	78.88
G3/D_2O	20.5	62.75
G4/D_2O	22.52	60.40
G5/D_2O	24.11	41.94

[a] Total weight content of fluorine in polymer [b] Calculated using ^{19}F NMR integral ratio divided by total fluorine concentration, normalized to G2/MeOD integral.

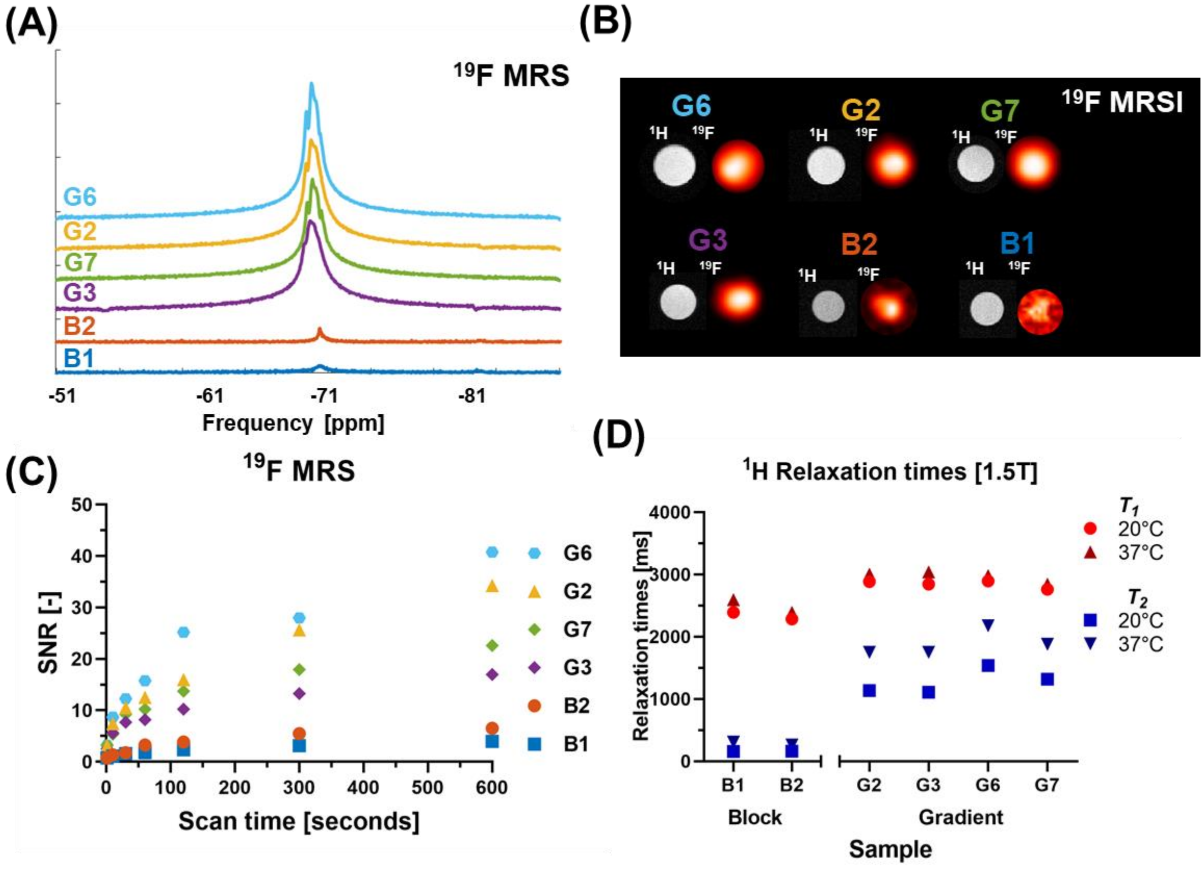

Figure S5. ^{19}F MRS (A) ^{19}F spectra of gradient vs block copolymers (B) MR images of gradient and block copolymers (C) Signal-to-noise ratio of the selected polymers (D) ^{1}H relaxation times [T_1, T_2]

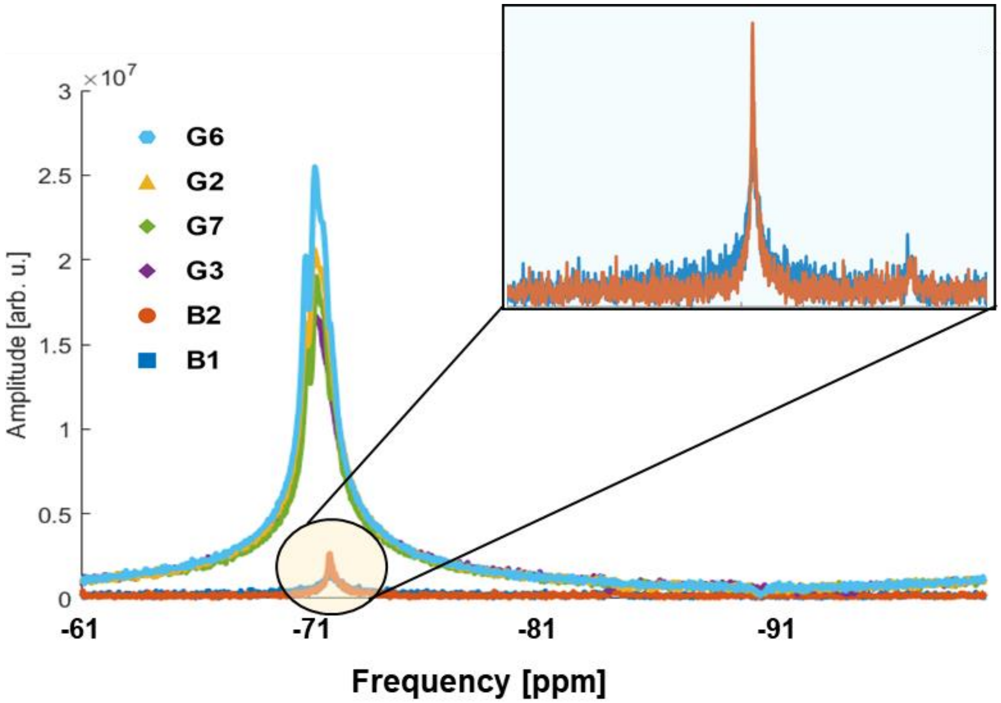

Figure S6. ^{19}F MRI (7T) spectra of copolymer nanoparticles in water.

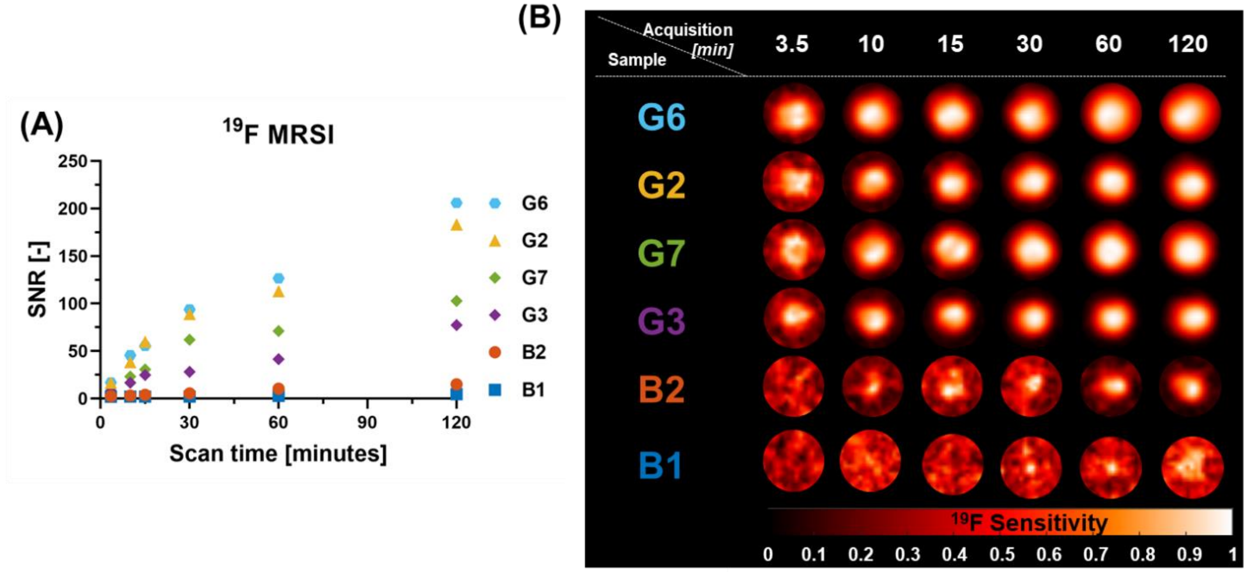

Figure S7. ^{19}F SNR and MR images of copolymer nanoparticles from 7T MRI.

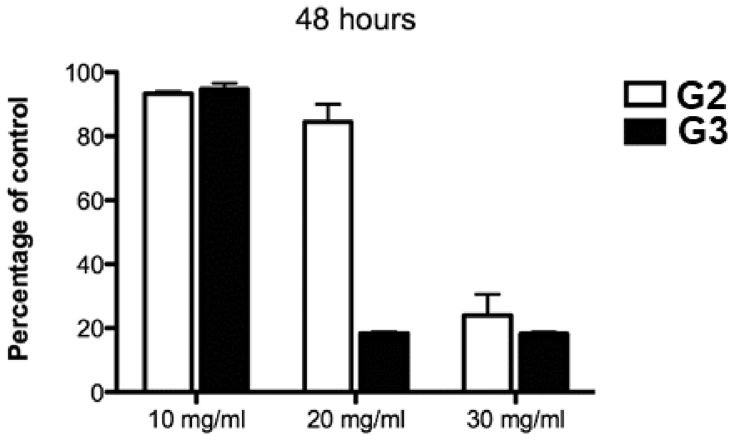

Figure S8. Impact on cell viability following exposure to self-assembled ^{19}F MRI tracers G2 and G3. Absorbance values were normalized, with the control cells set at 100 %. Treated cells underwent labeling for 48 hours using contrast based on fluorinated particles at concentrations of 10, 20, and 30 mg/ml. The data represent the mean of independent experiments ± standard error of the mean (SEM).

Kinetic Monte Carlo simulation code

The following simplified VBA code for visualization of repeating unit distribution in copolymers was used as a macro in Microsoft Excel software. The simulation has been performed for 250 chains on ($[PEGMA]_0$:$[TFEAM]_0$:$[CTCPA]_0$ = 50:200:1 statistical copolymerization with reactivity ratios r_{PEGMA} = 2.07, r_{TFEAM} = 0.16

```
Sub CopolymerizationSimulation()
    Dim rA As Double
    Dim rB As Double
    rA = 2.07 ' Reactivity ratio of monomer A
    rB = 0.16 ' Reactivity ratio of monomer B

    Dim amountA As Double
    Dim amountB As Double
    amountA = 50
    amountB = 200

    Dim chainLength As Integer
    chainLength = 250

    Dim numSimulations As Integer
    numSimulations = 100

    Dim ws As Worksheet
    Set ws = ThisWorkbook.Sheets("Sheet1")
```

```
    ws.Cells.Clear

    Randomize

    Dim sim As Integer, unit As Integer
    Dim currentAmountA As Double, currentAmountB As Double
    Dim totalAmount As Double, moleFractionA As Double, moleFractionB As
Double
    Dim randNum As Double, probA As Double, probB As Double
    Dim lastMonomer As String

    For sim = 1 To numSimulations
        currentAmountA = amountA
        currentAmountB = amountB
        lastMonomer = "A"

        For unit = 1 To chainLength
            totalAmount = currentAmountA + currentAmountB
            moleFractionA = currentAmountA / totalAmount
            moleFractionB = currentAmountB / totalAmount

            randNum = Rnd

            If lastMonomer = "A" Then
                probA = (rA * moleFractionA) / (rA * moleFractionA +
moleFractionB)
                probB = 1 - probA
            Else
                probB = (rB * moleFractionB) / (rB * moleFractionB +
moleFractionA)
                probA = 1 - probB
            End If

            If randNum < probA And currentAmountA > 0 Then
                ws.Cells(sim, unit).Value = "A"
                lastMonomer = "A"
                currentAmountA = currentAmountA - 1
            ElseIf currentAmountB > 0 Then
                ws.Cells(sim, unit).Value = "B"
                lastMonomer = "B"
                currentAmountB = currentAmountB - 1
            End If
        Next unit
    Next sim
End Sub
```
